# Natural pigments: innovative extraction technologies and their potential application in health and food industries

**DOI:** 10.3389/fphar.2024.1507108

**Published:** 2025-01-08

**Authors:** Ayu Masyita, Gemala Hardinasinta, Ayun Dwi Astuti, Firdayani Firdayani, Dian Mayasari, Aki Hori, Ira Nur Ainun Nisha, Firzan Nainu, Takayuki Kuraishi

**Affiliations:** ^1^ Research Center for Vaccine and Drugs, Research Organization for Health, National Research and Innovation Agency (BRIN), Cibinong Bogor, Indonesia; ^2^ Department of Agricultural Engineering, Faculty of Agricultural, Hasanuddin University, Makassar, Indonesia; ^3^ Faculty of Medicine, Hasanuddin University, Makassar, Indonesia; ^4^ Department of Pharmacy, Faculty of Pharmacy, Universitas Indonesia, Depok, Indonesia; ^5^ Faculty of Pharmacy, Institute of Medical, Pharmaceutical and Health Sciences, Kanazawa University, Kanazawa, Japan; ^6^ Department of Biological Sciences, Faculty of Teacher Training and Education, Muslim Maros University, Maros, Indonesia; ^7^ Department of Pharmacy, Faculty of Pharmacy, Hasanuddin University, Makassar, Indonesia

**Keywords:** natural pigments, extraction, antioxidant, anticancer, food colorants

## Abstract

Natural pigments, or natural colorants, are frequently utilized in the food industry due to their diverse functional and nutritional attributes. Beyond their color properties, these pigments possess several biological activities, including antioxidant, anti-inflammatory, anticancer, antibacterial, and neuroprotective effects, as well as benefits for eye health. This review aims to provide a timely overview of the potential of natural pigments in the pharmaceutical, medical, and food industries. Special emphasis is placed on emerging technologies for natural pigment extraction (thermal technologies, non-thermal technologies, and supercritical fluid extraction), their pharmacological effects, and their potential application in intelligent food packaging and as food colorants. Natural pigments show several pharmaceutical prospects. For example, delphinidin (30 µM) significantly inhibited the growth of three cancer cell lines (B16-F10, EO771, and RM1) by at least 90% after 48 h. Furthermore, as an antioxidant agent, fucoxanthin at the highest concentration (50 μg/mL) significantly increased the ratio of glutathione to glutathione disulfide (*p* < 0.05). In the food industry, natural pigments have been used to improve the nutritional value of food without significantly altering the sensory experience. Moreover, the use of natural pH-sensitive pigments as food freshness indicators in intelligent food packaging is a cutting-edge technological advancement. This innovation could provide useful information to consumers, increase shelf life, and assist in evaluating the quality of packaged food by observing color variations over time. However, the use of natural pigments presents certain challenges, particularly regarding their stability and higher production costs compared to synthetic pigments. This situation underscores the need for further investigation into alternative pigment sources and improved stabilization methods. The instability of these natural pigments emphasizes their tendency to degrade and change color when exposed to various external conditions, including light, oxygen, temperature fluctuations, pH levels, and interactions with other substances in the food matrix.

## Graphical Abstract

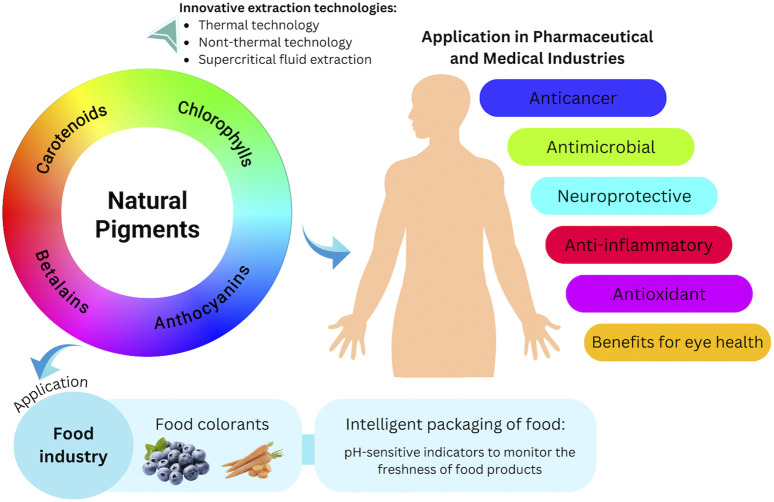

## 1 Introduction

Natural pigments are organic substances that impart the rich and diverse colors seen in many fruits, vegetables, flowers, and various other living organisms (Singh et al., 2023). These pigments are generally categorized into four main groups: anthocyanins, carotenoids, chlorophylls, and betalains (Magalhães et al., 2024). While they are primarily known for their contribution to the color of plants, these natural pigments also act as bioactive substances that may provide various health advantages. Diets rich in anthocyanins, for example, have been linked to enhanced heart health, better cognitive function, and improved vision (Wallace, 2011). Carotenoids are beneficial for boosting the immune system and promoting skin health, largely due to their antioxidant effects (Nabi et al., 2020). Additionally, carotenoids play a protective role in maintaining eye health, with evidence suggesting they help prevent age-related macular degeneration (Milani et al., 2017). Research on chlorophylls has highlighted their potential for detoxification, as they can bind to harmful carcinogens and possibly lower cancer risk (Martins et al., 2023; Sun et al., 2024). Lastly, betalains are noted for their potent antioxidant and anti-inflammatory actions, which have been associated with reduced oxidative damage and better cardiovascular health (Moreno-Ley et al., 2021).

Recently, there has been a marked increase in consumer demand for products containing natural colors. This trend has been driven by growing health and environmental concerns, leading to the widespread adoption of natural colorants as alternatives to synthetic pigments (Ghosh et al., 2022). Although the food industry has traditionally relied on synthetic colorants for their stability, vivid colors, and low production costs, the use of natural food pigments is progressively gaining traction. This shift is largely due to evolving consumer preferences and heightened awareness of the potential health risks and environmental consequences associated with synthetic pigments (Novais et al., 2022). Given their significant nutritional value and health-promoting properties, natural pigments are increasingly regarded as functional food ingredients. They not only enhance the sensory qualities of food products but also serve to mask undesirable attributes or improve the overall natural characteristics of food (Magalhães et al., 2024).

The extraction methods for natural pigments are evolving to enhance both product quality and extraction efficiency. The goal is to reduce extraction time and minimize solvent use compared to conventional methods. Furthermore, there are significant concerns regarding the environmental impact of toxic residues from organic solvents, as well as the safety of the final products derived from these methods (Panda and Manickam, 2019). Ensuring safety is crucial, especially when natural pigments are intended for use in the food, pharmaceutical, and cosmetic sectors. Therefore, current extraction processes are increasingly directed toward the use of novel technologies. These novel technologies are generally classified into advanced thermal methods, such as microwave, ohmic heating, and radiofrequency heating (Kubo et al., 2020), and non-thermal methods, such as pulsed electric fields, high pressure, ultrasound, and cavitation-based extraction (Panda and Manickam, 2019). These technologies can overcome the limitations of traditional extraction methods by reducing extraction time and solvent use, while also increasing the extraction yield of natural pigments, including anthocyanins and chlorophyll (Guo et al., 2019; Hsieh-Lo et al., 2020; Kutlu et al., 2021; Lefebvre et al., 2020).

Although natural pigments offer numerous benefits, they face several challenges that limit their broader application, particularly in food products. These challenges include issues with low bioavailability and stability (Nabi et al., 2023). However, encapsulation techniques have emerged as an effective solution to these problems, as they help protect the pigments from degradation. By encapsulating these compounds, their stability and bioactivity are enhanced, which in turn improves their potential health benefits (Ghosh et al., 2022).

In this context, and in light of the information mentioned above, further research in this area is essential. This review aims to provide an updated summary of innovative methods for extracting natural pigments while expanding our understanding of their value and potential applications across various fields, including food, medicine, and pharmaceuticals. Through a comprehensive analysis, we seek to enhance our knowledge of natural pigments and emphasize their promising therapeutic potential.

Several recent review articles (2021–2024) have explored the health potential of natural pigments; however, they primarily offer broad overviews without focusing on specific metabolites. For example, Magalhães et al. (2024) highlighted that anthocyanins are recognized for their significant health benefits, including anticancer properties linked to both chemopreventive and chemoprotective effects, as well as antioxidant and anti-inflammatory activities. Similarly Lu et al. (2021), found that carotenoids positively impact various reactive oxygen species (ROS)-related diseases, such as cardiovascular disease, osteoporosis, cancer, and myocardial infarction in smokers.

Therefore, the originality of this review lies in its focus on the biological activities of natural pigments and their metabolites, with particular emphasis on their anticancer, antioxidant, antimicrobial, anti-inflammatory, and neuroprotective properties, along with their positive impact on eye health. Furthermore, this paper explores the biotransformation and bioavailability of natural pigments, which are underexplored aspects of this field. Ultimately, this work aims to lay the groundwork for future developments in natural pigment-based drug discovery and their application in the food industry.

## 2 Methods

Research articles and reviews on natural pigments were gathered using the search engines Google Scholar, PubMed, Scopus, and SpringerLink. The search was based on a specific set of keywords: (“natural pigments” OR “anthocyanins” OR “carotenoids” OR “chlorophylls” OR “betalains”) AND (“extractions” OR “biotransformation” OR “anticancer activity” OR “antioxidant activity” OR “anti-inflammatory activity” OR “antimicrobial activity” OR “neuroprotective activity” OR “eye health” OR “stability” OR “food industry”).

During the preliminary screening, article titles and abstracts were manually reviewed to eliminate studies that were not pertinent to the topic. The selection criteria included: studies published in English, documents published between 2000 and 2024, availability of full text, and the presence of the terms “natural pigments” or their metabolites in the titles and/or abstracts. Exclusion criteria included: studies lacking details on chemical composition, extraction methods, biotransformation, biological effects, stability or application in food industry; records without full-text access; and articles published before 2000. In total, 244 articles were included in this study.

## 3 Classification of natural pigments

### 3.1 Anthocyanins

Anthocyanins are glucosides of anthocyanidins, a type of water-soluble pigment synthesized via the phenylpropanoid pathway (Mattioli et al., 2020). Structurally, anthocyanins are formed by 2-phenylchromenylium (a flavylium cation), which links methoxyl (−OCH₃) and/or hydroxyl (−OH) groups, along with one or more sugars (Al-Khayri et al., 2022). Anthocyanins are extensively found in the fruits, flowers, and vegetables of many plants, including fig (*Ficus carica* L.), juçara (*Euterpe edulis* Mart.), blackberry (*Rubus fruticosus* L.), grumixama (*Eugenia brasiliensis* Lam.), grapes (*Vitis vinifera* L.), blueberry (*Vaccinium myrtillus* L.), and petals of saffron (*Crocus sativus* L.) (Cerezo et al., 2020). These metabolites exhibit various colors such as blue, purple, and red, depending on their concentration and the complementary light absorption of chlorophyll (Bendokas et al., 2019).

**FIGURE 1:**
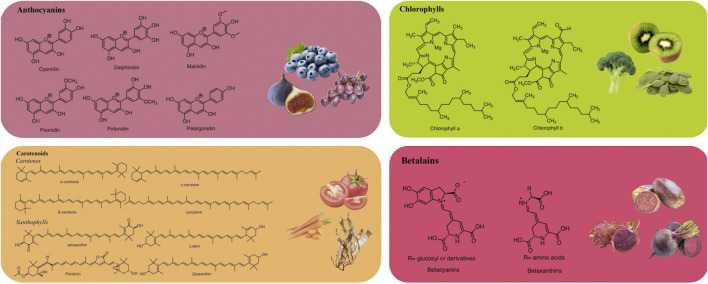
Representative chemical structures of natural pigments.

Anthocyanins are commonly utilized as natural colorants (Enaru et al., 2021). Nevertheless, pH, temperature, light, and structure all affect the color and stability of these metabolites (Khoo et al., 2017). For example, these pigments change from red at an acidic pH to blue at a basic pH. Additionally, the stability of anthocyanins is influenced by the presence of methoxyl or hydroxyl groups (Kang et al., 2021). Delphinidin, cyanidin, malvidin, pelargonidin, petunidin, and peonidin are the main anthocyanidins found in foods (Figure 1) (Enaru et al., 2021).

The total anthocyanin content in blueberries ranges from 85 to 270 mg/100 g fresh weight (FW). In blackberries, the primary anthocyanins are 3-glycoside derivatives of several flavonoids, including cyanidin, delphinidin, malvidin, petunidin, and peonidin. In contrast, cranberries have a lower total anthocyanin concentration, ranging from 25 to 100 mg/100 g FW. The dominant anthocyanins in cranberries are the 3-O-galactoside and 3-O-arabinoside forms of cyanidin and peonidin (Padmanabhan et al., 2016).

### 3.2 Carotenoids

Carotenoids (a type of fat-soluble pigment) are naturally found in algae, plants, animals, photosynthetic bacteria, and some species of fungi and archaea (Bhatt and Patel, 2020). These metabolites are tetraterpene pigments, which impart orange, purple, yellow, and red colors (Maoka, 2020). The intensity of their color is typically correlated with the quantity of carotenoids. Carotenoids are most abundant in fruits and vegetables. Their basic structures commonly consist of a 40-carbon backbone with eight isoprene units (Sun et al., 2022).

Structurally, carotenoids can be classified into two groups: carotenes and xanthophylls (Figure 1). Carotenes are hydrocarbons, including compounds like lycopene and β-carotene. In contrast, xanthophylls are derived from carotenes through the introduction of oxygen-containing functional groups such as hydroxyl, methoxy, carboxyl, keto, and epoxy. Prominent xanthophylls include lutein, β-cryptoxanthin, zeaxanthin, and fucoxanthin (Maoka, 2020; Nisar et al., 2015). Additionally, carotenoids can exist in an acyclic form, as in the case of lycopene, or exhibit various cyclic structures at one or both ends, similar to β-carotene. Due to the numerous double bonds in their molecular chains, carotenoids can adopt several cis/trans isomeric forms, although the all-trans isomer is the most stable and is predominantly found in nature (Molina et al., 2023). Martí et al. (2016) reported that tomatoes are a source of carotenoids, including lycopene, phytoene, phytofluene, β-carotene, γ-carotene, and δ-carotene, with concentrations ranging from 7.8 to 18.1 mg/100 g FW, 1.0–2.9 mg/100 g FW, 0.2–1.6 mg/100 g FW, 0.1–1.2 mg/100 g FW, 0.05–0.3 mg/100 g FW, and 0–0.2 mg/100 g FW, respectively.

### 3.3 Chlorophylls

Chlorophylls are naturally occurring green pigments found in algae, cyanobacteria, and several plants (Tanaka and Tanaka, 2019). Structurally, chlorophylls are complex molecules classified as porphyrins. They consist of four pyrrole rings and an additional isocyclic ring adjacent to the third pyrrole ring. These rings are connected by methylene bridges, with a magnesium atom at the center of the molecule. Additionally, in the fourth pyrrole ring, the propionic acid is esterified with a long-chain acyclic alcohol, typically phytol, which imparts a hydrophobic property to chlorophyll a (Molina et al., 2023; Vaňková et al., 2018). Chlorophylls are commonly found in two major forms: chlorophyll a and chlorophyll b (Figure 1), which differ at the 7-carbon position (Yilmaz and Gökmen, 2016). Chlorophyll a contains a methyl (–CH_3_) group, while chlorophyll b contains an aldehyde (–CHO) group. These structural differences result in different colors; chlorophyll a appears green-blue, while chlorophyll b appears green-yellow. The ratio of chlorophyll a to chlorophyll b in plants is typically 3:1 (Carillo et al., 2022).

Chlorophylls have relatively low stability due to their structural susceptibility to various factors that can alter their color characteristics. One of the most common reactions affecting chlorophyll stability is the replacement of the central magnesium ion with two hydrogen ions. This substitution causes a significant color change, as magnesium-containing derivatives appear green, whereas those lacking magnesium, such as pheophytins and pheophorbides, exhibit a brown coloration (Molina et al., 2023).

He et al. (2018) found that the concentrations of chlorophyll a and chlorophyll b in the green alga *Ulva prolifera* at 28°C were 3.2 ± 0.04 μg/mL and 1.90 ± 0.06 μg/mL, respectively. Furthermore, Šamec et al. (2021) examined the chlorophyll content in Brassica leafy vegetables. Their results showed that kale had chlorophyll a and chlorophyll b concentrations of 7.21 ± 0.19 μg/g dry weight (DW) and 3.50 ± 0.48 μg/g DW, respectively. White cabbage exhibited chlorophyll a and chlorophyll b concentrations of 4.69 ± 0.40 μg/g DW and 2.17 ± 0.87 μg/g DW, respectively, while Chinese cabbage contained chlorophyll a and chlorophyll b at concentrations of 4.67 ± 0.46 μg/g DW and 4.30 ± 0.53 μg/g DW, respectively.

### 3.4 Betalains

**FIGURE 2:**
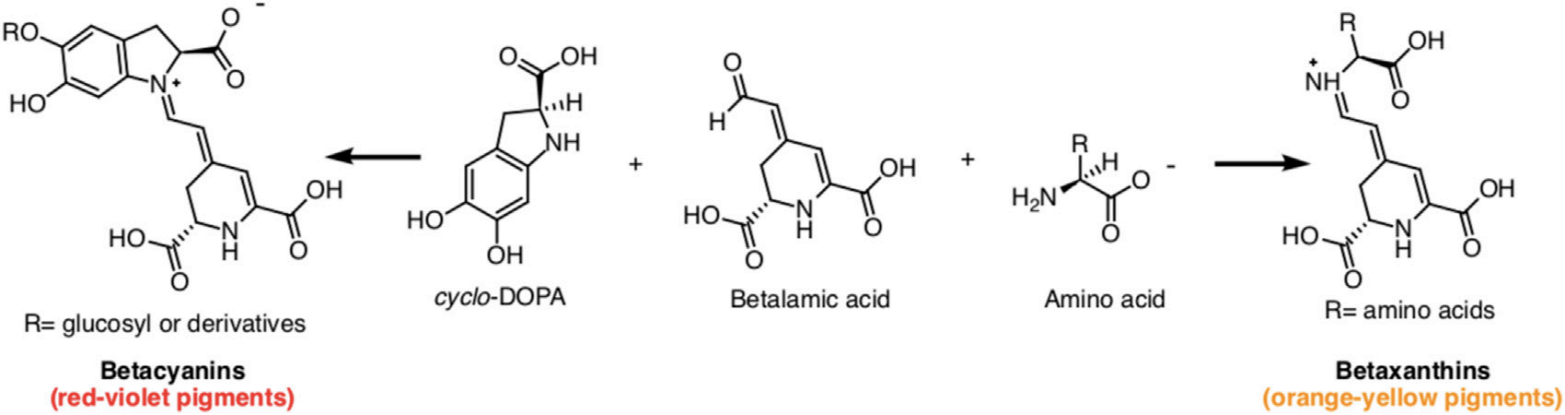
Betacyanins and betaxanthines chemical structures produced by condensing betalamic acid with cyclo-DOPA and amino acids, respectively.

Betalains, water-soluble nitrogenous pigments, are composed of betalamic acid (4-(2-oxoethylidene)-1,2,3,4-tetrahydropyridine-2,6-dicarboxylic acid) as their basic structure (Madadi et al., 2020). These pigments are classified into two groups: betaxanthins (yellow pigments) and betacyanins (violet pigments) (Figure 1) (Calva-Estrada et al., 2022). Cyclo-L-3,4-dihydroxyphenylalanine (cyclo-DOPA) or its glucosyl derivatives condense with betalamic acid to form betacyanins (Silva et al., 2020). Additionally, betalamic acid and amino metabolites condense to form betaxanthins (Figure 2) (Silva et al., 2020). Betalains are widespread in vegetables and fruits, with concentrations ranging from 13.81 to 2,252 mg/100 g in red dragon fruit (*Selenicereus monacanthus* (Lem.) D.R. Hunt), prickly pear (*Opuntia* spp.), and xoconostle (*Opuntia joconostle* F.A.C. Weber ex Diguet) (Kumar et al., 2020; Jiménez-Alvarado et al., 2015).

## 4 Emerging technologies in natural pigment extraction

The extraction of pigment from the plant can be optimized by utilizing various emerging technologies. These technologies simplify the extraction process, reducing used solvents and extraction time without compromising the extraction yield. In addition, emerging technology could also include novel techniques to obtain “green” solvents as a substitute for the ionic liquid used in extraction (Guo et al., 2019). This section will address the effect of several technologies in extracting natural pigment. These technologies could be classified into thermal and non-thermal technologies.

### 4.1 Thermal technology

#### 4.1.1 Ohmic heating

In the extraction process, thermal treatment is generally avoided due to its negative effect on thermal-sensitive metabolites, including natural pigment. However, novel thermal technologies including ohmic heating and microwave have been developed intensively in the past decades to overcome the drawbacks of conventional thermal treatments. Most studies revolve around the application of these technologies in processing food and beverages (Ferreira et al., 2019; Kuriya et al., 2020). These studies reported several quality parameters improvements in products treated with ohmic heating, including an increase in the reddish color of raspberry-flavored whey drink (Ferreira et al., 2019) and sensory attributes of dulce de leche (Kuriya et al., 2020). Those findings intrigue other studies to evaluate the viability of ohmic heating in the extraction process of bioactive and pigment metabolites by modifying the treatment parameters.

Ohmic heating provides rapid and uniform heating with a low energy consumption due to the absence of direct contact between the heating surface and the product (Pereira et al., 2020). The baseline of ohmic heating induces an electric current to a product with specific electrical conductivity. In ohmic heating, the product will behave as a resistance to the electric current flow, increasing temperature from inside the product. The increasing temperature will initiate cell wall disruption, which causes the metabolite to be extracted in the solvent. The heating process in ohmic is based on the principle of High-Temperature Short Time (HTST), where the heat generation occurs rapidly to increase the lethality of microbial and enzyme inactivation while preventing significant damage to bioactive metabolites.

The applied electric field increases plant tissue’s permeability, allowing faster diffusion of the metabolite into the liquid medium, particularly the low molecular weight metabolites such as anthocyanins. Pereira et al. (2020) further demonstrated that the HTST effect during ohmic heating pre-treatment (40°C–100°C, <20 s) increased the anthocyanin content extracted from grape skin from 756 to 1,349 μg/g DW. The intense treatment (100°C, 80 V/cm) provided higher anthocyanin content than the mild treatment (40°C, 20 min, 16 V/cm), proving that thermal and electrical treatment positively affects the anthocyanin extraction in grape skin solution.

Compared with conventional extraction methods, ohmic heating at specific processing conditions could increase natural pigment’s extraction yield. For instance, ohmic heating-assisted extraction of natural pigment from red beetroot resulted in a higher yield of betalain metabolite than conventional extraction at 40°C. The optimum conditions reported in the study were the application of voltage gradient at 17 V/cm and a frequency of 400 Hz at 40°C using aqueous ethanol as the extracting medium (Cabas and Icier, 2021). The study further reported that the yield of betacyanin and color changes (hue angle, ΔE, ΔC, chroma) increased as the voltage gradient increased. Higher L* and a* values were observed during ohmic heating using aqueous ethanol, indicating a brighter, red-colored extract. Darker extract characterized by a low L* (brightness) value can be achieved by applying the highest voltage gradient and the lowest frequency (Cabas and Icier, 2021).

Another study by Pagels et al. (2021) demonstrated that the ohmic extraction of cyanobacteria pigments resulted in a higher yield of carotenoids and phycobiliproteins than the homogenization method. Ohmic heating-assisted extraction obtained 41.59 ± 1.71 mg/g DW of carotenoid and 136.6 ± 10.0 mg/g DW of phycobiliproteins, which are 1.3 and 1.2-fold higher than the homogenization method, respectively. In addition, ohmic heating extraction using ethanol and water as the medium also provided higher antioxidant capacity, 8.04 ± 0.31 and 8.33 ± 0.31 mg TE/g DW, respectively. This research concluded that the optimum processing conditions were achieved at 70°C and a frequency of 20 kHz for 5 min (Pagels et al., 2021).

The extraction of anthocyanins from grape by-product using the mixture of water and citric acid (1 mg/mL) as a solvent resulted in an insignificant difference between the ohmic and conventional methods (acidified methanolic solution) (Coelho et al., 2021). Besides better extraction yield, other competitive advantages of ohmic heating are higher energy efficiency and the possibility of extraction without organic solvent, which could promote better environmental impact. Kuriya et al. (2020) reported a higher energy efficiency in the ohmic heating of dairy desserts compared to conventional heating. Lower energy consumption can be achieved at a higher voltage gradient (9.1 V/cm) because of a faster heating rate of 17.1°C/min, while the heating rate in conventional heating only reached 9.9°C/min.

#### 4.1.2 Microwave heating

The microwave-assisted extraction (MAE) mechanism lies in the effect of electromagnetic waves on polar molecules contained by the sample, which induce dipole rotation. The radiation of electromagnetic waves also charged ions inside the sample, transferring the energy and allowing ionic movement. This molecular movement generated heat and resulted in the evaporation of moisture trapped inside the plant’s cellular matrix (sample). The loss of moisture creates pressure inside the plant tissue and disrupts the cell structure. The rupture of the cell structure allows mass transfer, where the solvent diffuses into the plant matrix and leaches compounds into the extractant (Boateng, 2024; Nonglait and Gokhale, 2024; Usman et al., 2023).

Recent trends in utilizing microwave heating for metabolite extraction have revolved around optimizing said method (Bener et al., 2022; Doldolova et al., 2021; Lal et al., 2021; Martínez-Abad et al., 2020; Shukla et al., 2022; Sudha et al., 2024). Generally, combining high temperature or power with short irradiation time is deemed the best combination for MAE of plant materials (Zin et al., 2020). For instance, in the extraction of curcumin and antioxidants from turmeric, the extraction temperature plays a significant role in obtaining the highest extraction yields (Doldolova et al., 2021). The optimum operating conditions of MAE and other extraction technologies for various applications and their yields are reported in Table 1.

**TABLE 1:** Extraction of bioactive metabolites with various technologies.

Treatment	Evaluated metabolites	Optimum condition		Extraction yield	Reference
		Solvent	Processing parameters		
Ohmic heating	Anthocyanin	NaCl (0.1 mol/L)	Temp: 100°C Electric field: 80 V/cm Frequency: 25 kHz	1,349 μg/g DW	Pereira et al. (2020)
Ohmic heating	Betalain Betacyanins Betaxanthins	Aqueous ethanol	Temp: 40°C Electric field: 17 V/cm Frequency: 400 Hz	Betalain (%): 68.50 ± 1.46 Betacyanins (%): 67.77 ± 2.35 Betaxanthins (%): 69.5 ± 0.71	Cabas and Icier, (2021)
Ohmic heating	Carotenoid Phycobiliproteins	Ethanol	Temp: 70°C Frequency: 20 kHz Time: 5 min	Carotenoid: 41.59 ± 1.71 mg/g DW Phycobiliproteins: 136.6 ± 10.0 mg/g DW	Pagels et al. (2021)
MAE	Total Phenolic Content (TPC)	Ethanol (36%) Solvent/sample ratio 44 mL/g	Temperature: 130°C, Time: 39 min	TPC: 73.2 ± 3.8 mg GAE/g peel dm	Figueroa et al. (2021)
MAE	Total Anthocyanin Content (TAC), Total Phenolic Content (TPC) Rotal Flavonoid Content (TFC) Antioxidant Activity (AA)	Ethanol concentration (v/v) 60% Particle size: 100 μm (in 100 mL solvent)	Power: 63 W Time: 101 s	TAC: 10.5 mg LE/g dw TPC: 606.0 mg GAE/g dw TFC: 195.0 mg CE/g dw AA: 79.5% and 82.2%	Shukla et al. (2022)
MAE-NADES	Curcumin Contents (CC)	a. Fructose:choline chloride:water (2:5:5) b. Sucrose:choline chloride:water (1:4:4) c. Fructose:lactic acid:water (1:5:5) d. Sucrose:lactic acid:water (1:5:7) e. Lactic acid:choline chloride:water (1:1:2)	Temp: 64.7°C–71.8°C, Time: 15.4–21.6 min Solvent/solid ratio: 14.5–16.5 mL/0.2 g	CC: 37.5%–41.4%	Doldolova et al. (2021)
Microwave-assisted hot aqueous extraction	Bixin and Norbixin	Seed/water ratio 1:1 (w/v)	Temp: 60°C Time: 30 min	Bixin: 0.581% Norbixin: 2.965%	Sudha et al. (2024)
Pulsed Electric Field (PEF) - MAE	Pectin	Pectin extract:ethanol 1:4 v/v liquid-to-liquid ratio	PEF strength: 11.99 kV/cm PEF treatment time: 5.47 min MAE power density: 647.30 W/g MAE time: 5 min	18.24%	Lal et al. (2021)
PEF	Total Phenolic (TP) Anthocyanin	—	Electric field: 1318 V/cm 315 pulses Pulse width: 100 m	TP: 19% Anthocyanin: 6%	Gagneten et al. (2019)
PEF	Betanin Vulgaxanthin	Phosphate buffer pH: 6.5	Electric field: 4.38 kV/cm Pulse number: 20 pulses	Betanin: 329% Vulgaxanthin: 244%	Nowacka et al. (2019)
High Hydrostatic Pressure (HHP)	Lycopene Flavonoid	Hexane (60%)	Pressure: 450 MPa Temp: 20°C Time: 10 min	Lycopene: 2.01 ± 0.09 mg QE/100 g FW Flavonoid: 21.52 ± 0.09 mg QE/g FW	Briones-Labarca et al. (2019)
HHP	Betanin Total Phenolic (TP) Total Flavonoid (TF)	Water Solid/water ratio 1:2 (g/mL)	Pressure: 500 MPa Temp: 10°C Time: 3 min	Betanin: 66.60–236.12 mg/100 g TP: 2,114.94 mg GAE/100 g in hull (804.67 mg/100 g in whole grain (352.87 mg/100 g in de-hulled seed TF: (910.27–1,011.73 mg QE/100 g in hull	Sun et al. (2019)
NPC-Ultrasound (UAE)	Six Main Flavonoid	Ethanol (72%) Solid/liquid ratio 25:1 mL/g	Intensity: 0.347 W/cm^2^ Pressure: 0.07 MPa Temp: 60°C Time: 16 min	Rutin: 125.17 mg/g Nicotiflorin: 15.02 mg/g Narcissin: 25.61 mg/g Quercetin: 51.89 mg/g Kaempferol: 4.32 mg/g Isorhamnetin: 6.30 mg/g	Wang et al. (2020)
NPC-UAE	Total Phenols (TP) Total Flavonoids (TF) Total Procyanidins (TPA)	Ethanol concentration (v/v) 68.61% Solid/liquid ratio 1:30 g/mL	Intensity: 0.36 W/cm^2^ Pressure: 0.07 Pa Temp: 50°C Time: 15 min	TP: 352.078 GAE mg/g DW TF: 113.426 RE mg/g DW TPA: 212.722 CE mg/g DW	Wang et al. (2018)
NPC-Deep Eutectic Solvents (DES	Total Phenolic	Choline Chloride (ChCl) and Ethylene Glycol (EG) Molar ratio 1:3 Volume ratio 6:4 Solid/liquid ratio 1:15	Pressure: 0.1 MPa Time: 25 min	90.33 ± 1.89 mg/g	Cao et al. (2019)
NPC-Tea Saponin (TS) Surfactant	Seven Target Flavonoid	60% Ethanol containing 0.5% (w/v) TS Solid/liquid ratio: 53 mL/g	Pressure: -0.07 MPa Temp: 61°C Time: 16 min	19.80 mg/g	Cui et al. (2020)
Superficial Fluid Extraction (SFE)	Carotenoid Rosmarinic acid Chlorophyll (*a* and *b*)	Carotenoid: 100% CO_2_ Rosmarinic acid: 10% EtOH/Water 50/50 v/v Chlorophyll: 30% EtOH	Carotenoid: 25°C and 20 MPa Rosmarinic acid: 25°C and 10 MPa Chlorophyll: 25°C and 10 MPa	Carotenoid: 53 mg/g Rosmarinic acid: 78 mg/g Chlorophyll: 100 mg/g	Lefebvre et al. (2021)
SFE	Chlorophyll a (Chl a) Total Carotenoid (TC)	Supercritical CO_2_ (SC – CO_2_)	Temp: 333 K Flow rate: 0.15 kg/h Pressure: 300 bar (Chl a) 400 bar	Chl a: 0.34 μg/g TC: 0.78 μg/g	Mouahid et al. (2020)
Radio Frequency Extraction (RF)	Total Phenolic Content (TPC)	Ethanol (70%) Liquid/solid ratio 20 mg/mL	Temp: 68.7°C Time: 10 min Conveyor speed: 2.5 m/s Electrode gap: 180 mm	Clitoria ternatea (CTE): 18.90 mg GAE/mL Hibiscus rosa sinensis (HBE): 14.03 mg GAE/mL	Kochadai et al. (2022)
Radio frequency-assisted enzymatic extraction (RF-E)	Anthocyanin	Cellulase/pectinase ratio 1:1 (w/w) 0.1% Ethanol (50%) Liquid/solid ratio 50 mL/g pH 4	Temp: 40°C Time: 10 min Electrode gap: 5 cm	50.87 mg C3G/100 g powder	Jiang et al. (2020)
RF	Pectin	Citric acid pH 2.2	Temp: 88°C Time: 19 min	11.24% ± 0.69%	Zheng et al. (2021)

Microwave heating is often paired with Natural deep eutectic solvents (NADES) to promote an eco-friendly extraction process (Bener et al., 2022; Doldolova et al., 2021; Tapia-Quirós et al., 2024). Since the main advantage of MAE is the rapid and efficient extraction process with a smaller sample volume requirement, it is desirable to couple it with other green methods, such as NASES, which could provide efficient extraction yield (Tapia-Quirós et al., 2024). Bener et al. (2022) reported that hazelnut samples extracted using natural solvents of choline chloride:1,2-propylene glycol (CC-PG) with MAE resulted in a higher antioxidant capacity than those of ethanolic extracts.

Other studies have reported the eminence of MAE in providing a more efficient extraction process. MAE extensively decreased the pigment extraction time from walnut green peel from 80 to 1 min with an extraction yield of 19.95% (Wang et al., 2022). Furthermore, the extraction of pectin polysaccharide from jackfruit waste assisted with the combination of pulsed electric fields (PEF) and (MAE) improved the yield (18.24%) and lowered the energy (0.0986 kW-h) consumption (Lal et al., 2021).

#### 4.1.3 Radio frequency heating

Radio frequency (RF) is an extension of microwave heating with a longer wavelength that allows deep penetration strength and enables RF to heat thicker and bigger sample sizes (Gao et al., 2023; Kochadai et al., 2022). Recently, RF has been utilized in the extraction process of metabolite, such as extraction of TPC (Fan et al., 2023; Kochadai et al., 2022), total flavonoid (Song et al., 2022), anthocyanin (Jiang et al., 2020), galacturonic acid (Zheng et al., 2021), also lignan, chlorophyll, and carotenoid content (Fan et al., 2023). Based on those studies, extraction with RF resulted in a higher extraction yield than hot water, acidified ethanol, and enzymatic (pectinase and cellulase) assisted extractions (Jiang et al., 2020). Meanwhile, flavonoid content showed no significant difference between RF and enzymatic-assisted (EA) extraction (Song et al., 2022). Detailed information about the processing parameters, extraction yield, and the used solvent is listed in Table 1.

### 4.2 Non-thermal technology

#### 4.2.1 Pulsed electric field (PEF)

The pulsed electric field is a non-thermal processing technology that applies short electric pulses to a product that promotes cell electroporation. This process occurs due to cell wall disruption formed by the applied pulses, which allow metabolites to be extracted into the liquid medium (Jaeschke et al., 2019). Compared to other conventional extraction methods, the primary advantages of PEF are its mild processing conditions (temperature), short processing time, and lower energy consumption (Gagneten et al., 2019). The electric field strength is the main factor affecting the effectiveness of PEF treatment (López-Gámez et al., 2021; Nowacka et al., 2019).

An investigation to improve the extraction of bioactive substances from blackcurrant reported that at optimum conditions, PEF treatment could enhance the total phenolic and anthocyanin content by up to 19% and 6%, respectively. A 1318 V/cm electric field strength and 315 pulses were used to achieve the optimum condition, resulting in a cumulative energy intake of 30 ± 2 kJ/kg (Gagneten et al., 2019). However, total phenolic and antioxidant activity decreased beyond the optimum conditions (>1300 V/cm). This study indicates that bioactive molecules may be degraded by the strong electrical energy used during PEF treatment. It is essential to point out that this study had a relatively wider pulse width (100 m) compared to other studies, which used 4 μs (López-Gámez et al., 2021), 1 μs (Jaeschke et al., 2019), and 10 μs (Nowacka et al., 2019). In addition, a slight temperature increase of 5°C was observed after the PEF treatment. The Joule effect formed by applying electric current to a product resulted in an increase in temperature with a linear correlation with electric current (Gagneten et al., 2019).

Nowacka et al. (2019) evaluate the effect of PEF treatment on the extraction of betanin and vulgaxanthin from beetroot. This study demonstrated that PEF treatment of beetroot cylinders at 4.38 kV/cm enhanced both pigments by up to 329% and 244%, respectively. However, in terms of beetroot pulp, applying PEF decreased the vulgaxanthin content, with a significant effect observed at a higher electric field (6.25 kV/cm). Although the betanin content increased in beetroot pulp, the changes were insignificant. The authors stated that the degradation of vulgaxanthin could be affected by the physicochemical characteristics of this pigment, where vulgaxanthin is more prone to temperature and pH change than betanin. It is important to note that an increase in product temperature for up to 9.1°C was observed after PEF treatment. The difference in the sample phase (solid and liquid) could also contribute to the degradation of this pigment. The liquid phase (beetroot pulp) could facilitate faster pigment extraction to the medium, resulting in a longer exposure time to a higher temperature for vulgaxanthin.

#### 4.2.2 High pressure-assisted extraction

Pressure-based technology such as High Hydrostatic Pressure (HHP) is commonly used for the sterilization and pasteurization of food and beverages due to the lethality of this technology to inactivate microorganisms and enzymes (Al-Ghamdi et al., 2020). The pressure used for this method falls in the range of 100–1,000 MPa. To reduce product contamination, this method uses the action of high pressure to disrupt the cell membranes of microorganisms. This effect can also be utilized to extract bioactive metabolites since cell wall disruption of the product could increase cell permeability, which leads to a high release of these metabolites (Briones-Labarca et al., 2019).

The application of HHP for the extraction process was conducted by Briones-Labarca et al. (2019), who evaluated the changes in lycopene concentrations in tomato pulp during the HHP extraction. The optimum processing conditions were achieved by applying pressure of 450 MPa and 60% hexane concentrations. This study reported that lycopene and flavonoid content increased as the pressure and solvent concentration increased.

A study comparing the effect of HHP and conventional heating method on the betanin content of the Djulis plant (*Chenopodium formosanum* Koidz.) stated that higher levels of betanin were observed in the HHP-treated samples, ranging from 66.60 to 236.12 mg/100 g (Sun et al., 2019). This study also evaluated the effect of temperature during the HHP treatment using two different temperatures of 10°C and 30°C. The study demonstrated that the content of betanin pigment showed better retention of betanin at lower temperatures. The highest betanin content was obtained in the following treatment parameters: 500 MPa, 10°C, and 3 min. Although HHP treatment showed better retention of betanin content, this method caused significant degradation of betanin compared to the untreated sample (88.52 and 252.68 mg/100 g).

Based on previous research, temperature negatively affects pigment extraction by HHP. In addition, applying high pressure could increase the product temperature due to adiabatic heating, which could further damage the thermo-labile metabolite (Al-Ghamdi et al., 2020). Therefore, evaluating the cumulative impact resulting from the combination of high pressure and temperature during HHP processing is essential.

#### 4.2.3 Negative pressure cavitation

The main principle of Negative Pressure Cavitation (NPC) is inducing nitrogen into the extraction chamber by decreasing the pressure inside (negative pressure). This process produced air bubbles and simultaneously generated rapid turbulence between the solid-liquid-gas inside the system. The cavitation and turbulence effect further leads to cell wall disintegration allowing faster mass transfer of metabolites into the solvent (Wang et al., 2020).

The NPC method is often coupled with other extraction methods. Recent studies (2018–2022) primarily reported the utilization of NPC to extract total phenolic and flavonoids. This technique mainly assisted with applying “green solvent” to enhance the extraction yield and reduce the use of toxic substances. Table 1 lists several studies that evaluated the extraction of bioactive metabolites assisted with the NPC method.

Regarding the Negative Pressure Cavitation-Ultrasound Assisted Extraction (NPC-UAE) method, the extraction yield of bioactive metabolites increased following the increase in negative pressure, ultrasonic power, temperature, time, and solid/liquid ratio. However, after reaching specific conditions, an increase in these parameters caused the metabolites to degrade (Wang et al., 2020; Wang et al., 2018). The negative pressure applied in the extraction of flavonoids is the critical parameter in enhancing the amount of hyperin, hibifolin, isoquercetin, myricetin, quercetin-3′-O-glucoside, quercetin and rutin (Cui et al., 2020).

A study that compared three different extraction methods (NPC, microwave, and ultrasound) found that the highest extraction kinetic (k value) was obtained using the NPC method. The k value of NPC was significantly higher (0.338 min^-1^) compared to microwave (0.247 min^-1^) and ultrasound (0.220 min^-1^) (Cui et al., 2020). The higher k value in the NPC method indicates that the reaction reaches an equilibrium state faster than other methods, resulting in a greater extraction yield in a shorter time.

The ability of the NPC method to increase the extraction yield of phenolic and flavonoids implies that this method could also be used to extract natural pigment from the plant. The important thing to underline is determining the optimum conditions for different pigment types since each metabolite could behave differently under extraction parameters.

### 4.3 Supercritical fluid extraction

Selectivity is another essential aspect in extracting natural pigment to improve the purity of the metabolite, increase the concentration of targeted metabolites, or avoid undesired metabolites. These objectives can be achieved using Supercritical Fluid Extraction (SFE) (Lefebvre et al., 2021). Supercritical fluid has low viscosity and relatively high diffusivity. By altering the pressure and temperature, these characteristics are easily modified to the desired solubility therefore allowing the extraction process to aim for a specific metabolite. The high diffusivity of supercritical fluid increases the mass transport of the metabolite to the solvent (Al Jitan et al., 2018).

Carbon dioxide (CO_2_) is the prevalent extraction supercritical fluid. Another advantage of SFE is that this fluid is easily mixed with other solvents to obtain a modifier that allows selective extraction. For instance, the extraction of pigment from Rosemary using SFE reported that rich carotenoid extract could be obtained using pure CO_2_ at 25°C and the pressure of 20 MPa. In contrast, extraction of chlorophylls reached the optimum condition by using 30% ethanol as a modifier (Lefebvre et al., 2021). Extraction of carotenoid and chlorophylls from *Nannochloropsis salina* D.J.Hibberd. microalgae also reported similar conditions where CO_2_ would result in better carotenoid retention. At the same time, ethanol co-solvent increased both recoveries of chlorophylls and carotenoids by 67.6% and 12%, respectively (Mouahid et al., 2020).

Pressure plays a significant role in the optimization of the SFE method. The extraction of pigments from microalgae species, *Nannochloropsis maritima* D.J.Hibberd., showed that the effect of pressure during SFE treatment varies among pigments. Under 200 bar, pressure significantly affects total carotenoids’ recovery but is insignificant to chlorophylls. Meanwhile, above 200 bar, a linear correlation between pressure and both pigment’s recovery was observed (Mouahid et al., 2020). In addition, pressure could also affect the retention time needed to extract polyphenols. Lefebvre et al. (2021) reported that the extraction time of rosmarinic acid was reduced to 5.1 min from the initial time of 7.5 min by increasing the pressure from 10 to 20 MPa. In this case, increasing the pressure resulted in increased solubility of the modifier (10% EtOH:Water), allowing faster extraction.

## 5 Biotransformation of natural pigments

Biotransformation encompasses two main concepts: bioavailability and bioaccessibility. Before nutraceuticals are absorbed in the intestine, they must undergo several processes to reach enterocytes. This process is referred to as bioaccessibility (Toti et al., 2018). The bioavailability of natural pigments is an important aspect that requires further study, as pharmacokinetic and pharmacodynamic data on natural pigment metabolites remain very limited. Additionally, natural pigments can provide biological effects at low concentrations (González-Ponce et al., 2018).

Betalains are glycosylated flavonoids derived from a limited number of sources, including amaranth, pitaya, red beet, cactus pear, Swiss chard, and some tubers. These natural pigments have low bioavailability, typically around 1% of the amount consumed. They can be absorbed into the systemic circulation without undergoing hydrolysis in the small intestine and reach peak plasma levels within three hours. Urinary excretion reveals several newly formed metabolites whose biological activities are still unknown (González-Ponce et al., 2018).

The bioavailability and bioaccessibility of chlorophyll are influenced by two factors: the structure and the matrix of the chlorophyll (Chen and Roca, 2018). Isolated chlorophyll extracts exhibit the highest bioaccessibility compared to wet ultrasonicated biomass and whole dried biomass, particularly when ingested from *Scenedesmus obliquus*. The uptake of chlorophyll derivatives, such as hydroxypheophytin a, pheophytin a’, pheophytin a, and pheophytin b, from aqueous micellar fractions in Caco-2 cells showed a dominant uptake of up to 92%. Caco-2 cells are a human epithelial cell line widely used as a model for the intestinal epithelial barrier (Lea, 2015). For effective intestinal absorption, combining chlorophyll with micelles in an aqueous phase is necessary (Chen and Roca, 2018).

Carotenoids are found in human foods and can be detected in blood (plasma or serum) after ingestion (Maoka, 2020). Due to their fat solubility, differences in adiposity between men and women may also affect the bioavailability of carotenoids (Hayhoe et al., 2017). Additionally, the type of carotenoid can influence its bioavailability; for example, the bioavailability of most *trans*-β-carotene is greater than that of the cis form, while lycopene is better absorbed in its *cis* form than in *trans* (Priyadarshani, 2017). Daily consumption of paprika for 3 months demonstrated varying bioavailability of xanthine metabolites: β-cryptoxanthin accumulated at higher levels than zeaxanthin, while capsanthin was not detectable in plasma. The absorption of capsanthin was very slow, indicating a need for further study regarding its dosage, absorption, and accumulation in plasma (Umigai et al., 2018).

Anthocyanins are rapidly metabolized and easily oxidized, which is why they are poorly bioavailable. Anthocyanins found in Rosella flowers exhibited better stability at 70°C and in an acidic environment compared to weakly acidic conditions. The degradation of Rosella anthocyanins follows a first-order kinetic reaction, with greater degradation occurring in weakly acidic environments and at temperatures above 70°C (Wu et al., 2018). In the small intestine, anthocyanins are hydrolyzed to anthocyanidins, the aglycone forms of anthocyanins, producing the aldehyde metabolite phloroglucinolaldehyde and a phenolic acid after being metabolized by gut microflora, without breaking the B-ring of anthocyanins (Winter and Bickford, 2019).

The intestinal microflora generally digests glycosylated anthocyanins, but interactions with macromolecules in the large intestine can also degrade some of these compounds (Li et al., 2019). Cyanidin-3-rutinoside, cyanidin-3-glucoside, and delphinidin-3-rutinoside are anthocyanin metabolites that differ in their metabolic processes. When placed in simulated large intestine conditions, the three metabolites obtained from mulberry fruit were monitored for changes and products of bacterial-dependent metabolism. Cyanidin-3-glucoside disappeared after six hours, producing metabolites such as protocatechuic acid, p-coumaric acid, vanillic acid, and 2,4,6-trihydroxybenzaldehyde, similar to cyanidin-3-rutinoside, but with a longer metabolism time of eight hours. Meanwhile, delphinidin-3-rutinoside produced metabolites such as syringic acid, 2,4,6-trihydroxybenzaldehyde, and gallic acid after eight hours. The metabolism of different anthocyanin metabolites can yield various products, each of which may have distinct biological effects on humans (Chen Y. et al., 2017).

## 6 Roles of natural pigments in health

**FIGURE 3:**
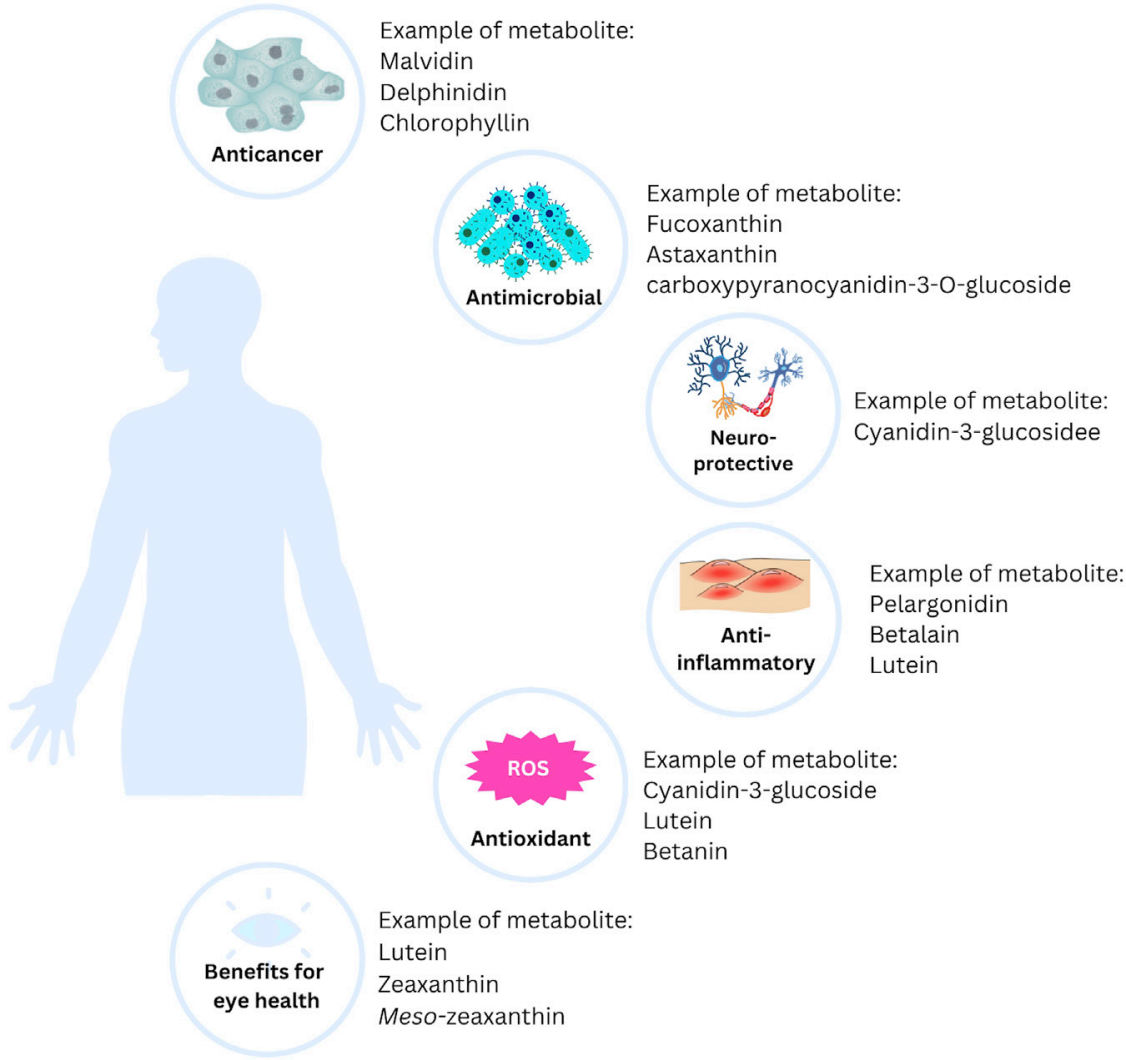
The potential health benefits of natural pigments.

Most of the natural pigments found in fruits, vegetables, and microorganisms can be beneficial to health, particularly in the treatment of chronic and degenerative diseases. Free radicals, one of the targets in treating various diseases, can be kept under control with natural pigments. Additionally, long-term use of natural pigments is generally safe and, therefore, more acceptable as a treatment option (Leong et al., 2017). Here are some of the benefits of natural pigments in health (Figure 3).

### 6.1 Anticancer activity

The anticancer potentials of natural pigments have been reported extensively in recent years (Table 2). Lu et al. (2024) studied the effect of zeaxanthin on the adhesion, proliferation, invasion, and migration of human glioblastoma (GBM) cell lines. They found that zeaxanthin may prevent GBM angiogenesis and tumor growth by downregulating a series of oncogenic signaling pathways. Zeaxanthin inhibited the activation of the VEGFR2 kinase pathway produced by VEGF and reduced the expression of p-AKT, p-ERK, p-STAT3, and FAK in U251 cells (Lu et al., 2024). Similarly, Shin et al. (2020) studied the ability of astaxanthin (AXT) as a novel anticancer agent. They reported that low doses (4–8 μM) of AXT decreased the expression of the tumor protein p53 and increased the levels of cyclin-dependent kinase (CDK) 2 and p-Cdk2/3 in the astroglioma cell lines U251-MG (Shin et al., 2020).

**TABLE 2:** Mechanism of anticancer activity of natural pigment metabolite.

Metabolite	Target	Concentration	Method	Mechanism of action	Reference
Malvidin	Dalton’s lymphoma ascites in mice	5 mg/kg bw and 10 mg/kg bw	*In vivo*	Decrease in tumor volume, levels of aspartate aminotransferase (AST), alanine aminotransferase (ALT), alkaline phosphatase (ALP), gamma-glutamyl transferase (GGT) and cellular glutathione (GSH) in tumor cells.	Sakthivel et al. (2020)
Malvidin-3-galactoside	Hepatocellular carcinoma (HepG2 cells)	0, 50, 100, and 200 g/mL	*In vitro*	Inhibition of MMP pathways by decreased the expression of MMP-2 and MMP-9.	Lin et al. (2020)
Cyanidin 3-O-glucoside	BALB/cByJNarl mice, H661 cells injected in mice	5 mg/kg/d	*In vitro* and *in vivo*	Increased apoptosis, decreased levels of IL-1β, TNF-α, C-reactive protein, IL-6), cyclooxygenase-2 and NF-κB proteins; increased inhibition of NF-κB kinase α mRNA; and downregulated transforming growth factor-β, CD44, epidermal growth factor receptor, and vascular endothelial growth factor.	Wu et al. (2022)
Delphinidin	B16-F10 melanoma cells injected subcutaneously to mice, C57BL/6 N mice, primary human umbilical vein endothelial cells (HUVECs)	10 mg/kg intraperitonial	*In vitro* and *in vivo*	Inhibited cell proliferation through VEGFR2, MAPK, PI3K signaling at the transcriptional level as well as PDE2 inhibition.	Keravis et al. (2015)
Delphinidin	RM-1 (CRL-3310), E0771 (CRL-3461), B16-F10 Luc2 (CRL-6475-LUC2), C57BL/6 mice	50 mg/kg and 100 mg/kg	*In vivo*	Inhibited hyaluronidase activity and suppressed melanoma metastasis in mice	McGuire et al. (2024)
Delphinidin	SKOV3 cell	50 μM and 75 μM	*In vitro*	Inhibited BDNF-stimulated expression of MMP-2 and MMP-9. Also, the BDNF-induced increase in SKOV3 ovarian cancer cells’ cell migration and invasion was markedly reduced by delphinidin.	Lim et al. (2017)
Delphinidin	A549 (human lung adenocarcinoma cells)	1, 10, 20, 40, and 80 μM	*In vitro*	Inhibited angiogenesis by suppressing expression of HIF-1α and VEGF.	Kim et al. (2016)
Lutein	Human gastric cancer cell line AGS, MKN-74, MKN-1 and SNU-668	5, 10, or 20 μM	*In vitro*	Activated NADPH oxidase to produce ROS and induced apoptosis in gastric cancer AGS cells.	Eom et al. (2023)
Astaxanthin	Astroglioma cell lines U251-MG and T98G	4, 8, 10, 20, and 40 µM	*In vitro*	Triggered the hormesis of astroglioma cells by down-regulating the p53 and up-regulating the cyclin-dependent kinase.	Shin et al. (2020)
Zeaxanthin	Human gliobalastoma cell lines U87, U251, BALB/c nude mice	5, 10, 20, 40, 80 µM	*In vitro* and *in vivo*	Inhibited glioblastoma angiogenesis and tumor growth by downregulated a cascade of oncogenic signaling pathways.	Lu et al. (2024)
Chlorophyllin	HeLa cell	0.05–16 μg/mL	*In vitro*	Induced apoptosis through oxidative stress.	Heo et al. (2023)
Betanin	Human colorectal cancer cell lines (Caco-2 and HT-29)	20, 40, 60, 80, and 100 µM	*In vitro*	Inhibited the growth of HT-29 and Caco-2 cells through inducing apoptosis.	Saber et al. (2023)

Furthermore, it has been reported by Kim et al. (2016) that delphinidin exhibits anticancer activity against A549 lung cancer cells. When 80 µM delphinidin was administered, EGF-induced angiogenesis was almost entirely inhibited and significantly decreased. Additionally, the EGF-induced rise in hemoglobin content was dose-dependently decreased by delphinidin (Kim et al., 2016).

Some natural pigments besides having anticancer activity can also function as co-treatments that can increase the effectiveness of anticancer drugs and/or reduce their side effects. For example, astaxanthin at a concentration of 5 μg/mL can increase MCF-7 breast cancer cell proliferation by 14%, while concentrations of 10 and 15 μg/mL can inhibit cell proliferation by 1% and 19%, respectively. However, when astaxanthin 5 μg/mL was combined with carbendazim at doses of 15 and 30 μM, the antiproliferative effect was increased by 8% and 16%, respectively (Atalay et al., 2019). The search for new anticancer metabolites also targets mortalin, a protein that can bind to p53 and prevent p53 from entering the nucleus. This causes inhibition of cell cycle arrest and apoptosis in cancer cells. Fucoxanthin has been reported to suppress mortalin transcription. In addition, fucoxanthin causes a decrease in cell proliferation, cell metastasis, and survival of cancer cells but is safe for normal cells (Garg et al., 2019). Fucoxanthin can also be an adjuvant therapy with doxorubicin in breast cancer. The combination of 1 µM of doxorubicin and 10 µM of fucoxanthin against MDA-MB-231 cells could significantly increase the number of apoptotic cells and decrease cell proliferation (Malhão et al., 2021). Bacterially produced bio-pigments mostly have medicinal properties. Astaxanthin from *Pontibacter kolensis* sp. nov. inhibits the growth of the MCF-7 cell line (Pachaiyappan et al., 2021).

Anthocyanins can trigger apoptosis and inhibit the proliferation of HT29 cells in human colon cancer. In a mouse colon tumor model, anthocyanins can reduce PI3K protein expression, increasing Bcl-2/Bax- and caspase-dependent apoptotic pathways and decreasing colorectal cancer growth (Zhao X. et al., 2019).

BIU87 cell growth in bladder cancer can be inhibited at a rate of 36.28% with anthocyanins obtained from purple sweet potato at a concentration of 800 μg/mL through an apoptotic mechanism. Apoptosis increases with increasing concentration, which indicates that the anticancer activity of purple sweet potato anthocyanins is dose-dependent (Li et al., 2018). The substituent on B-rings of anthocyanins affects their anticancer activity. Orto-dihydroxy phenyl on B-rings of anthocyanins can inhibit the growth and metastasis of cancer cells starting with the inhibition of inflammation by suppression of COX-2 and iNOS and expression via the PI3K/Akt andNF-κB pathway at the initial stage. Anthocyanins with orto-dihydroxyphenyl on B-rings also can regulate the expression of cancer-associated genes, thereby triggering cell cycle arrest and DNA repair by inhibiting RTK activity and playing a role in MAPK and AP-1 pathways. This mechanism occurs at the formation stage. Anthocyanins mediated by ROS and JNK/p38-MAPK can activate caspases resulting in cancer cell apoptosis at the development stage (Lin et al., 2017).

Some cancer proteins such as IGF-1R kinase proteins, CDK-2, and CDK-6 bind to anthocyanidins, namely petunidin, peonidin, malvidin, pelargonidin, delphinidin, and cyanidin, thus anthocyanidins are promising metabolites for the development of cancer drugs (Sravani et al., 2021).

Crocin and crocetin, carotenoids from Iranian saffron, inhibit SOD activity by scavenging superoxide radicals and affecting the copper-binding site, respectively. This inhibition was observed in breast cancer cells using docking analysis (Hashemi et al., 2020). Crocin and crocetin also have antimetastatic effects on 4T1 cells, triple negative metastatic breast cancer cells, through inhibition of cell mobility, migration, and invasion, also reduce adhesion to the extracellular matrix (Arzi et al., 2018).

### 6.2 Antioxidant activity

Recent decades have demonstrated that natural pigments possess significant antioxidant properties (Lu et al., 2021). Chlorophyllin, is a water-soluble salt containing copper and sodium that is a homologue of the common green pigment chlorophyll and has been reported to exert an antioxidant effect. Chlorophyllin boosted the activities of antioxidant enzymes (glutathione reductase, glutathione peroxidase, and glucose-6-phosphate dehydrogenase) (Ozcan et al., 2021). Moreover, it has been reported by Yu et al. (2020) that cyanidin-3-glucoside (C3G) has been shown to have a hepatoprotective impact for liver damage caused by oxidative stress because of its antioxidant effect. C3G increases the activity of antioxidant enzymes and upregulating the Nrf2-antioxidant pathway (Yu et al., 2020).

The role of carotenoids in disease cannot be separated from their antioxidant abilities (Rodriguez-Amaya, 2019). Lycopene is a carotenoid that has the highest antioxidant activity compared to other carotenoids (Costa-Rodrigues et al., 2018). Astaxanthin which is a C-C double chain conjugated with olefins can bind ROS and free radicals (Zhao T. et al., 2019) with a mechanism of action to form epoxides, the same as -Carotene and Zeaxanthin (Nishino et al., 2017). Fucoxanthin from *Phaeodactylum tricornutum* Bohlin, in a dose-dependent manner, can increase the ratio of reduced to oxidized glutathione in HeLa cells by up to 3.3 times (Neumann et al., 2019).

Anthocyanins have potential for treating neurodegenerative diseases because they can modulate various aspects of the disease, particularly the antioxidant pathway (Winter and Bickford, 2019). Chlorogenic acid and cyanidin are the most common anthocyanins found in mahonia fruit extracts, which exhibit antioxidant activity that is twofold higher than that of the phenolic fraction (Coklar and Akbulut, 2017). The ethanol extract of blueberry contains delphinidin, petunidin, cyanidin, peonidin, and malvidin, which are believed to contribute to the antioxidant activity of the extract (Zhou et al., 2019). The anthocyanin content of black chokeberry is 930 mg/g dry weight and consists of four primary anthocyanins: cyanidin-3-O-galactoside, cyanidin-3-O-xyloside, cyanidin-3-O-glucoside, and cyanidin-3-O-arabinoside (Meng et al., 2019). Summarized *in vivo* studies on the antioxidant activity of natural pigments are presented in Table 3.

**TABLE 3:** Antioxidant effect of natural pigment evaluated in an *in vivo* model.

Metabolite	Doses	Animal model	Level of evidence	Effects	Reference
Malvidin	100 mg/kg via intraperitoneal	Rat	5	Inhibition of oxidative stress in kidney tissue	Emadi et al. (2020)
Cyanidin-3-glucoside	100, 200, 400 mg/kg orally	Rat	5	Inhibition of cell cytotoxicity, apoptosis, oxidative stress-induced H_2_O_2_	Yu et al. (2020)
Fucoxanthin	0.1% orally	Mice	5	Reduced *in vivo* oxidative stress	Iwasaki et al. (2012)
Betanin	20 mg/kg via intragastric gavage	Rat	5	Reduced hepatic malondialdehyde and increased superoxide dismutase, catalase, and glutathione peroxidase activities.	da Silva et al. (2019)
Betanin	25 and 100 mg/kg via intragastric gavage and intraperitoneal	Rat	5	Increased malondialdehyde and myeloperoxidase activity, also reduced superoxide dismutase	Han et al. (2015)
Lutein	50, 100 and 250 mg/kg	Mouse	5	Increased the activity of catalase, superoxide dismutase, glutathione reductase, glutathione, glutathione peroxidase and glutathione-S-transferase	Sindhu et al. (2010)

Level of evidence: level 5 (animal studies).

### 6.3 Anti-inflammatory activity

The anti-inflammatory activity of anthocyanins was obtained through inhibiting the activation of NF-κB signalling pathway (Duarte et al., 2018; Mirza et al., 2019; Nguyen et al., 2018), decreased serum levels of IFN-γ (Ouyang & O’Garra, 2019), NO (Duarte et al., 2018; Winter et al., 2017), TNF-α, the expression of TLR4 (Cui et al., 2018), and iNOS (Duarte et al., 2018), inhibited MAPKs signaling pathway (Zhang et al., 2020) and reduce the production of ROS (González-Ponce et al., 2018).

The anti-inflammatory of anthocyanins from *Dioscorea alata* L. tubers was investigated using a mouse model with inflammatory bowel disease. The disease activity index reduces significantly compared to negative control with 80 μg/kg body weight of anthocyanins fraction. The responses, such as body weight, fecal consistency, and fecal occult blood, were similar to the positive control, 5-aminosalicylic acid. The anthocyanins fraction can downregulate the pro-inflammatory cytokines such as TNF-α, IFN-γ, MPO, and iNOS (Chen T. et al., 2017). The main anthocyanin in strawberry fruit, namely pelargonidin-3-O-glucoside, can modulate IL-10 production at a dose of 0.08 mol/L (Amini et al., 2017). Anthocyanins extract from sour cherry fruit (*Prunus cerasus* L.) at a dose of 50 μM can decrease IL-6 and IL-8 in the inflammation model using the Caco-2 cell line (Nguyen et al., 2018). In addition, anthocyanins from red clover (*Trifolium pratense* L.) in a dose-dependent manner can inhibit macrophage cells from secreting TNF-α to RAW 264.7 cells (Lee et al., 2020).

Lycopene not only has the highest antioxidant activity among other carotenoids, but it also has anti-inflammatory activity (Thies et al., 2017) through production inhibition of pro-inflammatory cytokines (IL-6β, TNF-α, and IL-1β) (Liu et al., 2018). Bixin, a derivative carotenoid, from *Bixa orellana* L. seeds, can reduce edema and increase heat resistance on the paw of rats induced by 30 mg/kg of carrageenan orally. The anti-inflammatory mechanism of bixin is related to decreased migration of neutrophils (Pacheco et al., 2019). Combining carotenoids and other compounds can increase their antioxidant and anti-inflammatory activity. For example, 5 µM of fucoxanthin and rosmarinic can decrease apoptosis and ROS production, and downregulate the production of interleukin (IL)-1β, NLRP3, Caspase-1, and ASC. In addition, the pre-treated with the combination can increase the expression of antioxidant genes (HO-1 and Nrf2) in HaCat cells exposed to UV-B (Rodriguez-Luna et al., 2019). Table 4 illustrates the anti-inflammatory activity of natural pigments through *in vivo* evaluation.

**TABLE 4:** Anti-inflammatory activity of natural pigments through *in vivo* evaluation.

Metabolite	Animal model	Doses	Evidence level	Key findings	Reference
Malvidin	Sepsis-associated encephalopathy (SAE) mouse	5, 10, 20 mg/kg	5	Reduced ROS accumulation through activating the AMPK-α/UCP2 axis	Zhao et al. (2023)
Cyanidin	Osteoarthritis mouse	50 mg/kg	5	Activates Sirt6 thereby protecting cartilage from degradation	Jiang et al. (2019)
Delphinidin	Spinal cord injury (SCI)-induced inflammation in a rat model	40 and 200 mg/kg	5	Inhibits COX-2 activity, decreases PGE2 production and protein expression of AP-1 and p38-MAPK, and prevents NF-κB stimulation.	Wang et al. (2017)
Pelargonidin	Mice induced with lipopolysaccharide	0.2, 0,4 and 0.6 mg/kg	5	Reduced TNF-α or IL-6 production and activates NF-κB or ERK 1/2	Lee et al. (2019)
Pelargonidin	C57/BL6 mice	0.5–160 μM	5	Inhibited the IL-1β-induced ECM catabolism in chondrocytes and reduced the IL-1β-stimulated p-p65 overexpression and MMP13.	Zeng et al. (2023)
Betalain	Male Swiss or C57BL/6 mice	10–1,000 mg/kg	5	Lower production of pro-inflammatory cytokines	Martinez et al. (2020)
Lutein	Zebrafish	100 and 200 μg/mL	5	Reduced induced NO synthase expression and proinflammatory cytokine release	Kim et al. (2023)
Astaxanthin	Male *db/db* micee	200 ppm	5	Inhibited NF-κB activation, and decreased the expression levels of IL-1β, IL-6, F4/80, CCL2, and CXCL2 mRNA.	Kochi et al. (2014)
Astaxanthin	Male Syrian hamsters	5, 10, and 15 mg/kg	5	Inhibited NF-κB activation	Kavitha et al. (2013)
β-carotene	Male Sprague–Dawley rats	20, 40 and 80 mg/kg	5	Reduced the production of pro-inflammatory cytokines, including TNF-α, interleukin-1β, interleukin-18 and COX-2	Zhou et al. (2018)

### 6.4 Antimicrobial activity

There has been a significant increase in interest in discovering new antibacterial substances, particularly those derived from natural sources, such as plants and marine ecosystems (seaweeds, microalgae, and crustaceans) (Gomes et al., 2022). Seaweeds contain a wide array of pigment compositions that provide several secondary metabolites, including carotenoids, chlorophylls, and phycobiliproteins (PBPs). Additionally, the pigments in seaweeds also include phenolic metabolites, which are responsible for antibacterial activity. A metabolite consisting of phlorotannins, which have a low molecular weight and are extracted from the brown seaweed *Sargassum thunbergii*, caused *Vibrio parahaemolyticus* to lose the integrity of its cell walls and membrane (Besednova et al., 2020). By altering and destroying the cell membrane, *Fucus vesiculosus* L. [Fucaceae, Fucus], a brown seaweed, produced oligomeric phlorotannins (phloroglucinol) that had bacteriostatic effects on pathogenic *Streptococcus aureus* and *S. pneumoniae* (Bogolitsyn et al., 2019; Gomes et al., 2022). Research has indicated that carotenoids, particularly fucoxanthin, a pigment found in seaweeds, are effective against both Gram-negative and Gram-positive bacteria. The findings showed that carotenoids hindered various bacteria’s ability to thrive under aerobic conditions. Fucoxanthin was effective in this study against some Gram-positive bacteria, such as *S. aureus*, *S. agalactiae*, and *S. epidermidis*, but ineffective against Gram-negative bacteria, such as *Klebsiella oxytoca*, *Escherichia coli*, and *K. pneumoniae* (Karpiński and Adamczak, 2019).

Anthocyanins, another group of natural pigments, also exhibit antibacterial effects. An earlier study found that anthocyanins had an antibacterial effect on *S. aureus*, *E. coli*, *Pseudomonas aeruginosa*, and *Enterococcus faecalis*, which are resistant to vancomycin, with minimum inhibitory concentration (MIC) and maximum tolerated concentration (MTC) of 31.07 and 7.76 mg phenol/well, respectively (Côté et al., 2011). Moreover, anthocyanins demonstrated the strongest sensitivity to both *Listeria innocua* and *Aeromonas hydrophilia*, with MIC values of 50 and 40 mg/mL, respectively (Genskowsky et al., 2016).

Fucoxanthin, a natural xanthophyll pigment belonging to the carotenoid family, frequently acts against aerobic bacteria at low concentrations (10–250 μg/mL) (Karpiński et al., 2021). Rajauria and Abu-Ghannam (2013) reported that fucoxanthin also acts against *Listeria monocytogenes* (inhibition zone: 10.27 mm) at a concentration of 25 µg compound/disc. In a different study, Peraman and Nachimuthu (2019) found that the MIC values of fucoxanthin against fungi (*Aspergillus brasiliensis*, *A. fumigatus*, and *Candida albicans*) as well as bacteria were between 1,000 and 4,000 μg/mL. Furthermore, Šudomová et al. (2019) demonstrated that fucoxanthin showed very low MIC values against *Mycobacterium tuberculosis* (2.8–4.1 µM, or 1.85–2.7 μg/mL).

Similarly Contreras-Ortiz et al. (2017), evaluated the antimicrobial effect of astaxanthin. They found that the viability of *Trypanosoma cruzi* was reduced in an *in vitro* study with astaxanthin dosages of 200–300 μg/mL. In another study, Shanmugapriya et al. (2018) demonstrated that astaxanthin can act against a variety of bacteria when formulated as a nanoemulsion. The minimum inhibitory concentration (MIC) values for both Gram-positive and Gram-negative species ranged from 500 to 4,000 μg/mL.

Raspberry, blueberry, strawberry, and blackcurrant extracts are examples of anthocyanin-rich extracts that have shown efficacy against Gram-negative bacteria, but not Gram-positive bacteria. This finding could be explained by the differences in cell wall structure between Gram-positive and Gram-negative bacteria. The outer membranes of Gram-negative bacteria protect them from hydrophobic chemicals but not from hydrophilic ones (Mayasari et al., 2021a). The antimicrobial properties of extracts containing anthocyanins may result from a variety of processes and synergistic interactions between the phytochemicals present, including anthocyanins, phenolic acids, weak organic acids, and a mixture of different chemical forms that interact differently with Gram-positive and Gram-negative bacteria (Tarozzi et al., 2007).

Similar to how blackcurrant extract affects *Saccharomyces cerevisiae* and *E. coli* (benefiting their growth), but slows the growth of *S. aureus* and *E. faecium* (Werlein et al., 2005). Another study found that the anthocyanin metabolite carboxypyranocyanidin-3-O-glucoside prevents *P. aeruginosa* and *S. aureus* strains from producing biofilms in persistent wounds (Correia et al., 2021).

Anthocyanins found in red, purple, and blue vegetables and fruits are key biological compounds that help prevent microbial infections through various mechanisms (Gawai et al., 2017). They exhibit antibacterial activity through several processes, such as damaging the morphology of bacterial cells and compromising the integrity of the cell membrane, including the structure of the intracellular matrix and cell wall. Additionally, anthocyanins cause instability in the cytoplasmic membrane, inhibit extracellular microbial enzymes, and lead to the permeabilization of the plasma membrane (Burdulis et al., 2009). The antimicrobial effects of natural pigments from various sources are summarized in Table 5.

**TABLE 5:** Antimicrobial effect of extracts containing natural pigments.

Natural pigment	Source of pigment	Methods	Antimicrobial effects	Reference
Anthocyanins	Cranberry juice with distilled water	Agar well-diffusion method	12 mm inhibition zone against *Escherichia coli* (ATCC 25922)	Puišo et al. (2014)
	Pomegranate juice	Agar well-diffusion method	30 mm inhibition zone in lysogen medium for *Escherichia coli* with MIC value of 40 μg/μL.	Pagliarulo et al. (2016)
	Pomegranate juice	Agar well-diffusion method	MIC value of 10.75–12.5 mg/mL against *Salmonella* bacteria	Wafa et al. (2017)
	Water extract of Roselle	Disc diffusion method	13.63 ± 1.00 mm inhibition zone against *Escherichia coli* bacteria (ATCC 8739)	Jung et al. (2013)
	Ethanol extract of berries of murta	Disc diffusion method	20 mm inhibition zone against *Escherichia coli* bacteria	Junqueira-Gonçalves et al. (2015)
	80% aqueous methanolic leaf extract of *Withania somnifera*	Agar well-diffusion method	32.00 ± 0.75 mm inhibition zone for *Salmonella typhi* with MIC value of 6.25 mg/mL and 19.00 ± 1.48 mm against *Klebsiella pneumoniae*	Alam et al. (2012)
	Methanol extract of lowbush blueberry	Disc diffusion method	MIC of 34.75 mg/mL and MBC of 69.60 mg/mL against *Salmonella typhimurium*	Lacombe et al. (2012)
	Ethanol extract of European cranberry	Agar well-diffusion method	Ranging from 13.33 ± 0.47 to 22.00 ± 1.41 mm inhibition zone against *Salmonella typhimurium*.	Česonienė et al. (2009)
	Methanol extract of red cabbage	Disc diffusion method	20 ± 1.0 mm inhibition zone against *Escherichia coli* (ATCC 25922) with MIC value of 100 mg/mL and MBC value of 200 mg/mL	Hafidh et al. (2011)
	Ethanol extract of Kunth fruits	Disc diffusion method	Ranging from 25.2 to 34.0 mm inhibition zone against *Salmonella typhimurium* through TSA media	Llivisaca et al. (2018)
Carotenoids	*5. Rhodotorula glutinis* (Fresen.) F.C.Harrison extract using petroleum ether, n-hexane, ethanol, and acetone (25:25:50 v/v/v)	Broth microdilution method	MIC against *S.* Typhimurium isolates (17.0 μL/mL) and mean MIC against the *S. aureus* isolates (4.1 μL/mL).	Naisi et al. (2023)
	6. Methanol extract of bacteria symbionts *Virgibacillus salariu*s Hua et al.	Agar dilution method	The average clear zone diameter for MDR *E. coli* antibacterial test ranges from 0.770 cm to 0.915 cm with positive control 1.924 cm. Also, against MRSA had an average diameter ranging from 1.218 cm to 1.405 cm, and a positive control of 2.109 cm.	Kusmita et al. (2021)
Betacyanins	7. Red beet extract (*Beta vulgaris* L.	Agar dilution method	The higher concentration of the acid added increases the antimicrobial activity against *E. coli* and *S. aureus*.	Lembong and Utama (2019)
	Beetroot (*B. vulgaris* L.) extract	Agar dilution method	MIC of BE against *B. cereus* was 15 mg/mL.	Gong et al. (2023)

### 6.5 Neuroprotective activity

Anthocyanins are phenolic pigments that are both colored and water-soluble. These pigments are found in glycosylated forms and are responsible for the purple, red, and blue colors in fruits and vegetables. Scientific research has shown that anthocyanins possess antibacterial and neuroprotective properties, promote neurological health, support vision, and guard against some non-communicable diseases (Gawai et al., 2017). The therapeutic effects of anthocyanins in treating neurodegenerative conditions, such as Parkinson’s disease, include their anti-apoptotic, anti-neuroinflammatory, and antioxidant capabilities (Patek-Mohd et al., 2018).

Both *in vivo* and *in vitro* studies have been conducted to assess the neuroprotective effects of anthocyanin metabolites. An *in vitro* study demonstrated that cyanidin-3-glucoside and cyanidin have neuroprotective properties against oxidative stress induced by hydrogen peroxide in a human neuronal cell line (SH-SY5Y). The results showed that pre-treatment with cyanidin-3-glucoside and 100 µM cyanidin significantly enhanced antioxidant activity in both the cytosolic fraction and membrane of SH-SY5Y cells. Moreover, cyanidin dramatically increased the proportion of active mitochondria and prevented DNA fragmentation caused by hydrogen peroxide (Tarozzi et al., 2007).

Research suggests that anthocyanins may be effective in treating neurodegenerative diseases, such as ischemia, Alzheimer’s disease, Parkinson’s disease, and other forms of nerve damage (Airoldi et al., 2018; Jung and Kim, 2018). Anthocyanins in their glycosylated forms can cross the blood-brain barrier and enter the central nervous system (CNS), where they exert their biological effects. The neuroprotective activity of anthocyanins is linked to the transporter bili translocase, which primarily targets the vascular endothelium and subsequently the brain network (Aqil et al., 2014). Furthermore, the polyphenolic cationic structure of anthocyanins enables them to scavenge free radicals, reduce ROS production, and suggest their potential efficacy in disorders associated with neurodegenerative processes. Anthocyanins support the PI3K/Akt/HO-1 pathway in Alzheimer’s disease and regulate the endogenous antioxidant Nrf2/HO-1 pathway (Ali et al., 2018).

In APP/PS1 transgenic AD mice, anthocyanin (12 mg/kg i. p. over 30 days) significantly improved memory performance. Cyanidin-3-O-glucoside may also inhibit the formation of soluble amyloid-β 25–35 oligomers and associated neurotoxicity in the human nerve cell line SH-SY5Y (Tarozzi et al., 2007). Additionally, when microglial cells are exposed to a blueberry anthocyanin-rich extract, the expression of the inflammatory genes iNOX and COX is reduced (Bensalem et al., 2016). The neuroprotective effects of the natural pigment anthocyanin are summarized in Table 6.

**TABLE 6:** Neuroprotective effect of extract containing anthocyanins.

Source of anthocyanin	Method	Neuroprotective effect	Reference
Black soybean	*In vivo* (animal model: Spargue-Dawly rats, dose: 0.1 mg/mL)	Anthocyanin showed cellular levels of proapoptotic proteins were decreased and the cellular level of the antiapoptotic protein Bcl-2 was increased compared to treatment with ethanol only.	Ali Shah et al. (2013)
Kenyan purple tea	*In vivo* (animal model: female adult healthy Swiss white mice, dose: 200 mg/kg)	Kenyan purple tea showed neuroprotective effect with raised GSH levels and can cross the BBB reinforcing the brain capacity of antioxidant.	Rashid et al. (2014)
Tart cherries	*In vivo* (animal model: C57BL/6 mice subjected to permanent middle cerebral artery occlusion, dose: 2 mg/kg)	Infarction volume was reduced by 27% compared to vehicle-treated mice. Additionally, these mice had lower levels of brain superoxide after treatment with cyanidin-3-O-glucoside.	Min et al. (2011)
Blueberry and grape seed extracts	*In vitro* (cell line: MES23.5 mouse–rat hybrid dopaminergic cell, BV2 microglial cell)	Anthocyanins from blueberry and grape seed extracts rescued rotenone-induce defects in mitochondrial respiration in a dopaminergic cell line and attenuated nitrite releaser from microglial cells by lipopolysaccharides.	Strathearn et al. (2014)
Chokeberry fruit	*In vivo* (animal model: adult male Kunming mice, dose: 15 and 30 mg/kg)	Anthocyanins blocked age-associated cognitive decline and response capacity in senescence accelerated mice.	Wei et al. (2017)

### 6.6 Benefits for eye health

Age-related macular degeneration (AMD) is a vision impairment linked to aging, causing damage to the macula and central retina, resulting in symptoms like dark spots, distortion, visual field defects, and vision loss (Machida et al., 2020). The retinal macular area contains high levels of xanthophyll carotenoids, including lutein, zeaxanthin, and meso-zeaxanthin (a zeaxanthin isomer). These carotenoids, known as macular pigment, are believed to protect the retina and vision by acting as antioxidants and filtering blue light (Johnson et al., 2020).

However, studies have shown that the levels of carotenoids in the macula decline with age. Additionally, analysis of macular pigment optical density (MPOD) indicates that MPOD also decreases with age, highlighting the importance of increasing carotenoid levels in the macular region for maintaining eye health (Wilson et al., 2021). MPOD measures the concentrations of lutein and zeaxanthin in the macula, expressed in optical density units ranging from 0 to 1 (Kijlstra et al., 2012). To date, several clinical studies have examined the effects of lutein and zeaxanthin supplementation on MPOD (Table 7).

**TABLE 7:** Level of the evidence regarding the influence of lutein and zeaxanthin intake on macular pigment optical density (MPOD).

Dose	Criteria	Intervention (N)	Placebo (N)	Duration (months)	Type of study	Level of evidence	Result	Reference
10 mg Lutein (L), 2 mg zeaxanthin (Z)	Age, gender, BMI	40	20	6	RCT	2^1^	Supplementation with lutein and zeaxanthin led to elevated MPOD levels at all follow-up visits, showing significant increases by day 42 compared to the placebo group. Additionally, serum levels of lutein, zeaxanthin, and BDNF also rose. These improvements were linked to enhanced visual and cognitive performance, as well as reduced eye strain and fatigue in the children taking the LZ gummies.	Parekh et al. (2024)
12 mg Lutein, 0.6 mg zeaxanthin	Age, smoking status, spherical equivalent, axial length	52	—	10	RCT	2	The treated group showed a statistically significant increase in MPOD (*F* = 17.0, *p* < 0.001) and a reduction in mfERG ring 2 P1 latency (*F* = 3.69, *p* = 0.04).	Berrow et al. (2016)
5 mg Lutein, 1 mg zeaxanthin	Age, gender, BMI, smoking status, lens status	64	62	24	RCT	2	No notable enhancement in MPOD was observed with the Visucam^®^ 200 following carotenoid supplementation. Multiple regression analysis indicated that factors such as age, female gender, lens status, and the presence of AMD had a significant impact on MPOD measurements.	Azar et al. (2017)
22.33 mg Lutein, 4.70 mg zeaxanthin	Age, BMI, smoking status	8	5	3	RCT	2	MPOD showed a significant increase in the treatment group (*p* < 0.001) compared to the placebo. The results indicate that the central retinal deposition of zeaxanthin and mesozeaxanthin was effective and can be observed after a brief period of supplementation, particularly with higher daily doses of macular carotenoids (27.03 mg).	Stringham and Stringham (2016)
6 mg Lutein, 2 mg zeaxanthin, 60 mg anthocyanins	Age, amenorrhea, BMI	72	—	8	RCT	2	Neither the xanthophylls (lutein and zeaxanthin) nor the anthocyanins, alone or in combination, at the chosen doses, were able to enhance MPOD after 8 months of supplementation.	Olmedilla-Alonso et al. (2018)
20 mg Lutein, 2 mg zeaxanthin, 0.3 mg *meso-*zeaxanthin	Age, gender, smoking status, BMI	63	—	2	RCT	2	MPOD showed a significant increase at all eccentricities in the treated group (*p* < 0.05). The final serum concentrations of MZ were positively and significantly associated with the final MPOD values.	Thurnham et al. (2015)
22.33 mg Lutein, 4.70 mg zeaxanthin	Age, BMI, smoking status	25	10	6	RCT	2	MPOD showed a significant increase (*F* = 3.55; *p* = .023) in the treated group, with an increase of 0.12 OD compared to the placebo group.	Stringham et al. (2019)
15 mg Lutein, 3 mg zeaxanthin, 10 mg *meso-*zeaxanthin	Smoking status, pregnant status, history of diabetics, hypertension, and gastrointestinal disorders.	15	—	6	non-RCT	3	Carotenoid supplementation results in a notable increase in MPOD levels. From baseline to week 24, the average MPOD in the LM group rose by 0.064, increasing from 0.418 to 0.482.	Bone et al. (2020)
20 mg Zeaxanthin	White/Caucasian ethnic, age, BMI, intraocular pressure	18	—	4	Single-arm	3	After 4 months of daily supplementation with 20 mg of zeaxanthin in healthy individuals, MPOD showed a significant increase, and this effect persisted even after the washout period.	Iannaccone et al. (2016)
20 mg Lutein, 4 mg zeaxanthin	Age, gender, BMI, intraocular pressure	16	—	4	Single-arm	3	The total volume of MPOD inside 9° eccentricity had increased considerably by week 8, and this trend persisted until week 16 (*p* < 0.0001). These findings proved that a high-dose lutein/zeaxanthin supplement was beneficial for MPOD volume.	Obana et al. (2020)

Level of evidence: level 2 (prospective study with one or more randomized controlled trials, level 3 (prospective non-randomized controlled studies).

AMD, age-related macular degeneration; BDNF, brain derived neurotrophic factor; BMI, body mass index; mfERG, multifocal electroretinogram; MPOD, macular pigment optical density; RCT, randomized controlled trials.

Hammond et al. (2017) investigated the impact of lutein and zeaxanthin supplementation on MPOD. Sixty-two older adults were randomized into two groups: one receiving 10 mg of lutein and 2 mg of zeaxanthin (n = 42), and the other receiving a visually identical placebo (n = 20). Data from 51 participants (average age 73.7 years) were analyzed, revealing a significant increase in MPOD from baseline to the 12-month mark (M = 0.58, SD = 0.23; *p* < 0.03) in the active supplement group, while the placebo group showed no significant change over the year (Hammond et al., 2017).

In a separate study, Renzi-Hammond et al. (2017) investigated the impact of lutein and zeaxanthin supplementation on cognitive function in younger, healthy adults. They conducted a randomized, double-blind, placebo-controlled trial involving 51 participants aged 18 to 30, who were part of a larger research project on xanthophylls and cognitive performance. The subjects were divided into an active supplement group (n = 37) and a placebo group (n = 14). The findings showed that the supplement, which contained 10 mg of lutein and 2 mg of zeaxanthin, significantly increased MPOD over the year compared to the placebo (*p* < 0.001). Daily intake of lutein and zeaxanthin, along with the rise in MPOD, was linked to notable improvements in spatial memory (*p* < 0.04) (Renzi-Hammond et al., 2017).

## 7 Roles of natural pigments in food industry

The organoleptic properties of food play a significant role in its acceptance, selection, and ultimately consumption by individuals. Color stands out as one of the most visually appealing and attractive features (Novais et al., 2022). Since the 1850 s, synthetic coloring agents have been extensively used because they are easy to manufacture, cost-effective, highly efficient in providing vibrant colors, and require only small quantities (Azman et al., 2018). However, many synthetic colors, particularly those containing aromatic rings and azo functional groups, have been found to pose health risks, including hyperactivity, allergic reactions, and asthma attacks. As a result, there has been a gradual shift from synthetic colorants to naturally derived alternatives, driven by changing consumer attitudes and an increased demand for safer options (Singh et al., 2023). Pigments are materials with a broad range of colors, some of which are water-soluble, and are widely utilized in various industries. The non-toxic properties of pigments generated by many microbes make them suitable for use in dyes, food, medicine, cosmetics, and other industrial applications.

The FDA considers several variables when assessing the safety of a novel pigment or a new use for an existing pigment as an additive in food. These include the immediate and long-term effects of consumption, the substance’s composition and physical characteristics, the manufacturing process, its stability, the likelihood of exposure and consumption, and the accessibility of analytical techniques for determining its purity and concentration in food (FDA, 2023).

An essential goal of the food industry is the production of foods with appealing appearances. Natural food colors are becoming increasingly popular in food preparation, as some artificial color additives have been linked to adverse health effects (Azman et al., 2018). Food colorants are important in the food industry because they may hide undesirable qualities or enhance the inherent qualities of food items. Due to this, they may also be used for various specific purposes depending on their color. Examples include anthocyanins, which are common water-soluble flavonoids with pH-dependent colors ranging from red to blue. These anthocyanins are known for their bioactive properties, such as antioxidant, anti-inflammatory, and chemopreventive effects. Carotenoids, which are predominantly found in fruits and vegetables, are widely prized for their orange, yellow, and red colors, which enhance the flavor of meals and beverages. Betalains are another type of pigment that has emerged as a promising alternative to Red 40, a synthetic colorant known as Allura Red AC, which contains benzidine—a possible carcinogen for both humans and animals (Luzardo-Ocampo et al., 2021).

Purified colorants or natural pigments have been used to enhance the nutritional qualities of bread products without significantly affecting their sensory properties. A study by Abdel-Moemin (2016) found that cupcakes containing Rosella calyces (RC) extract received higher sensory ratings (*p* < 0.05) than the control cupcakes. Consuming 100 g of the RC cupcakes provided 465 mg of anthocyanins per 100 g (Abdel-Moemin, 2016). Additionally, López et al. (2019) demonstrated that incorporating *Arbutus unedo* L. extract, rich in cyanidin-3-O-glucoside, improved the color and antioxidant properties of wafers while leaving their nutritional profile largely unaffected (López et al., 2019).

Other natural products from fruit and vegetable waste can also serve as sources of coloring. Lycopene, for example, is extracted from tomato waste and used in cakes and biscuits (Eletr et al., 2017). Lycopene from tomato waste provides 300.85 mg of lycopene and 654.8 mg of total carotenoids per 100 g of fibrous pulp, which is free of peel and seeds.

To counteract any natural color loss that may occur during production and storage, milk and dairy products, like other foods, can be naturally colored. One of the most well-known natural colorants used in yogurt is derived from strawberries (red) and carrot juice (orange), both of which are employed in the yogurt industry to enhance the color and nutritional content of the product (Gawai et al., 2017).

Furthermore, cream cheese coloring agents derived from sea buckthorn fruit extracts (*Hippophae rhamnoides* L.) have been examined (Ghendov-Moşanu et al., 2020). Natural colorants have also been used in the formulations of other dairy products. For example, *Melastoma malabathricum* L. (“Senduduk” fruit) can double the amount of beta-carotene in jackfruit jam due to its antioxidant activity. Similarly, a previous study by Mayasari et al. (2021b) showed the antibacterial and antioxidant activity of *M. malabathricum* L. leaves. Lastly, the diverse applications of natural food pigments highlight their potential to be incorporated into various food systems beyond conventional formulas.

In addition, several recent studies have shown that natural pigments, which offer numerous advantages, have a promising future as they gain popularity and aid in the creation of intelligent packaging solutions (Oladzadabbasabadi et al., 2022). The structural instabilities of natural pigments can change color in different pH ranges, these pigments can be employed as harmless, natural pH indicators with great potential for usage in smart packaging (Ndwandwe et al., 2024). Colorimetric pH indicators are frequently employed in intelligent packaging as active tags to indicate pH changes through color changes (Pereira et al., 2015). Generally, when the food begins to deteriorate, the pH will change. This fact provides the scientific foundation for utilizing pH changes to evaluate food product quality (Pourjavaher et al., 2017).

For example, the betalains from *Bougainvillea glabra* Choisy were incorporated with potato starch to create a pH indicator film that allowed fish freshness to be tracked in real-time. The film’s color changes from pink to yellow, signifying the fish under observation losing quality (Naghdi et al., 2021). Similarly, the locust bean gum (LBG) film containing 5 wt% of the extracted anthocyanin from *Viola odorata* L. can be introduced as a smart packaging system to monitor the freshness of meat. The film showed color change from beige to light indigo after 6 days of storage at 4°C (Fathi et al., 2022). Table 8 shows the potential of natural pigments to be used in intelligent packaging as pH-sensitive indicators to monitor the freshness of food products.

**TABLE 8:** The potential of natural pigments to be used in intelligent packaging as pH-sensitive indicators to monitor the freshness of food products.

Natural pigment	Source of pigment	Polymer matrix	Concentration of pigment	Food	Storage condition	Range of color change	Results	Reference
Anthocyanins	Blueberry (*Vaccinium corymbosum* L.)	Poly-L-Lactic acid	0.05%, 0.1%, and 0.4% v/v	Mutton	0–72 h/25°C	Pink to colorless	Incorporating anthocyanin in film was able to monitor mutton freshness in real-time.	Sun et al. (2021)
	Red barberry (*Berberis vulgaris* L.)	Chitin nanofiber and methylcellulose	3% w/v	Fish	0–72 h/25°C	Crimson to pale pink	The film containing red barberry anthocyanin exhibited good antioxidant and antimicrobial. This film can protect and monitor the freshness of fish.	Alizadeh-sani et al. (2021)
	Butterfly pea (*Clitoria ternatea* L.)	Sago starch	5 mL of BP extract	Chicken	0–48 h/25°C	Blue to green	The developed film was effective in monitoring chicken freshness.	Ahmad et al. (2020)
	Roselle (*Hibiscus sabdariffa* L.)	Polyvinyl alcohol (PVA)/hydroxypropyl methylcellulose (HPMC)	0.12%, 0.18% and 0.24% w/v	Shrimp	0–10 d/4°C	Rose red to light yellow	The films’ improved functional qualities and freshness monitoring effects were facilitated by the closer contact between roselle anthocyanin extract (RAE) and the PVA/HPMC matrix as RAE contents increased.	Huang et al. (2021)
	Red apple pomace (*Malus pumila* Mill.)	Chitosan/nanosized TiO2	0.5%, 1%, 2.5%, and 5%	Salmon	0–48 h/25°C	Red to yellow	The film successfully as an indicator to monitor the freshness of salmon fillets.	Lan et al. (2021)
Betalains	Fresh red pitaya (*S. monacanthu)*		1,0 wt%	Shrimp	0–48 h/20°C	Red to colorless	Incorporating anthocyanin in film was successfully applied to monitor the freshness of shrimp.	Qin et al. (2020)
	Paperflower (*B. glabra* Choisy)	Potato starch	15% of betacyanin	Fish	0–16 d/4°C	Light pink to yellow	The betacyanin-starch film was able to monitor fish quality.	Naghdi et al. (2021)
	Fresh beets (*B. vulgaris* L.)	Banana starch	0.01 g for every 120 mL of filmogenic solution	Sausages	0–20 d/4°C	Not stated	Sausages covered with film kept their textural qualities for a longer time compared to the control.	Zamudio-Flores et al. (2015)
Carotenoids	β-carotene	LDPE/EVOH/PET	10 wt%	Peanuts	0–3 m/40°C	Yellow to slight amber	Incorporating β-carotene in the films delayed oxidative degradation, with an oxygen-absorbing capacity of 1.7 ± 0.3 mL O_2_/g.	Juan-Polo et al. (2022)
	Bixin	Polylactic acid	10 g/kg	Sunflower oil	0–16 d/40°C	Pale yellow to orange	The bixin-containing films reduced the amount of peroxides in sunflower oil (below 10 mEq/kg) and prevented food sensitive to oxygen from spoiling.	Stoll et al. (2023)
	β-carotene	Pectin/nanoclay	0.03%	Butter	0–90 d/-4°C	Orange to light yellow	The active packaged butter had the lowest microbial load, highest oxidative stability, and the least color change during storage.	Asdagh and Pirsa (2020)
Chlorophylls	Chlorophyll	Wheat gluten	2.5 and 5 mol/L	Sesame oil	0–8 d/25°C	Green to yellow	The films incorporated with chlorophyll increase shelf life and detect the expiration time of oil.	Chavoshizadeh et al. (2020)
	Green alga (*Caulerpa racemosa* (Forssk.) J.Agardh)	Alginate	0.5%, 1%, and 1.5%	Fish snacks	0–5 d/25°C	Not stated	The films incorporated with chlorophyll inhibit spoilage of fish snacks.	Dewi et al. (2022)

## 8 Technologies for improving pigment stability in food

Natural pigments have inferior stability compared to manufactured pigments due to their tendency to degrade in color quality. Understanding the physicochemical properties of pigments and their interactions with the food matrix is essential. Further studies are required to stabilize most of these pigments under varying temperature conditions and the typical pH range found in targeted food systems. It is crucial to implement technologies that enhance the stability of pigments in food. Chemical and environmental variables, including oxygen, pH, metal ions, light exposure, enzyme presence, and elevated temperatures, predominantly affect pigment stability (Cortez et al., 2017; Indrasti et al., 2018; Juric et al., 2020).

Adding co-pigment substances such as metals (Viera, Pérez-Gálvez and Roca, 2019), phenolic metabolites (Erşan et al., 2022), and polymers (de Boer et al., 2019) has been demonstrated as effective for enhancing and stabilizing natural pigments. For example, zinc and copper ions facilitate the synthesis of green metallochlorophylls in plants, leading to the re-greening of food products. However, the FDA’s maximum zinc concentration threshold of 75 ppm has proven ineffective. Encapsulation of zinc-chlorophylls using matrices such as gum arabic, maltodextrin, octenyl succinic anhydride (OSA)-modified starch, or whey proteins, along with the microencapsulation of raw chlorophyll extracts without salt treatment, is feasible (Viera, Pérez-Gálvez and Roca, 2019). Strawberry model solutions containing anthocyanins were treated with rooibos phenolics to improve their color and thermal stability, suggesting that rooibos could be used as a food ingredient to enhance the appearance of strawberries (Erşan et al., 2022). Biopolymers protect natural colorants from environmental factors such as high temperatures, oxidation, and photodegradation. Antioxidants and stabilizers are also encapsulated within these biopolymers, further increasing their stability (de Boer et al., 2019).

In addition to maintaining stability, nano-formulations and microencapsulation can increase solubility and facilitate the distribution of pigments into food matrices. Microencapsulation refers to the process of enclosing solids, gases, or liquids within sealed capsules ranging in size from millimeters to nanometers (Sen et al., 2019). Pigments that are susceptible to high temperatures should be encapsulated using freeze-drying or lyophilization. The absence of air and the low temperature in this process produce pigments that are resistant to oxidation and chemical changes (Enaru et al., 2021).

## 9 Conclusion and future remarks

This review enhances our knowledge of natural pigments, their pharmaceutical prospects, and their potential benefits in the food industry. The classification of natural pigments, extraction methods, biotransformation, pharmacological activities, and specific practical application cases in the food industry are systematically summarized. Innovative extraction technologies, such as thermal technology, non-thermal technology, and supercritical fluid extraction, offer promising ways to increase the yield and bioavailability of natural pigments.

Natural pigments have wide pharmaceutical and medical prospects, including anticancer, antioxidant, anti-inflammatory, antimicrobial, and neuroprotective effects, as well as benefits for eye health. Nevertheless, much remains unknown about their pharmacokinetic characteristics. Therefore, to determine the presence of active metabolites, comprehensive pharmacokinetic investigations of natural pigments should be conducted. The identification of these metabolites could yield important details about the pharmacological processes and bioactive forms of natural pigments.

Furthermore, these pigments are extensively utilized in the food industry as food additives to enhance the taste or impart color to food products. They also play a role in intelligent food packaging to monitor the freshness of food products. Due to their potential, increasing interest in natural pigments has been reported in the last decade.

However, there are still many gaps in the field of natural pigments. For example, emerging technologies for natural pigment extraction need to be improved for physicochemical feasibility, stability, and the ability to source these pigments from both traditional and novel origins. Research on natural pigments remains limited, especially regarding the mechanisms of action of their metabolites and their antimicrobial and neuroprotective effects. Future research may shift its focus to this area.

This discrepancy emphasizes the need for more thorough and rigorous research to increase the potential efficacy of natural pigments in disease management. Additionally, further research and development of practical and affordable methods are needed to scale up smart packaging technology based on natural pigments.
